# Stereology neuron counts correlate with deep learning estimates in the human hippocampal subregions

**DOI:** 10.1038/s41598-023-32903-y

**Published:** 2023-04-11

**Authors:** Jan Oltmer, Emma W. Rosenblum, Emily M. Williams, Jessica Roy, Josué Llamas-Rodriguez, Valentina Perosa, Samantha N. Champion, Matthew P. Frosch, Jean C. Augustinack

**Affiliations:** 1 Department of Radiology, Athinoula A. Martinos Center, Massachusetts General Hospital, Charlestown, MA USA; 2 Harvard Medical School, Boston, MA USA; 3 Department of Neurology, Massachusetts General Hospital, Harvard Medical School, J. Philip Kistler Stroke Research Center, Cambridge Str. 175, Suite 300, Boston, MA 02114 USA; 4 Department of Neurology, Otto-Von-Guericke University, Magdeburg, Germany; 5 Department of Neuropathology, Massachusetts General Hospital, Boston, MA USA

**Keywords:** Cell death in the nervous system, Neuroscience, Cellular neuroscience, Alzheimer's disease

## Abstract

Hippocampal subregions differ in specialization and vulnerability to cell death. Neuron death and hippocampal atrophy have been a marker for the progression of Alzheimer’s disease. Relatively few studies have examined neuronal loss in the human brain using stereology. We characterize an automated high-throughput deep learning pipeline to segment hippocampal pyramidal neurons, generate pyramidal neuron estimates within the human hippocampal subfields, and relate our results to stereology neuron counts. Based on seven cases and 168 partitions, we vet deep learning parameters to segment hippocampal pyramidal neurons from the background using the open-source CellPose algorithm, and show the automated removal of false-positive segmentations. There was no difference in Dice scores between neurons segmented by the deep learning pipeline and manual segmentations (Independent Samples *t-*Test: t(28) = 0.33, p = 0.742). Deep-learning neuron estimates strongly correlate with manual stereological counts per subregion (Spearman’s correlation (n = 9): r(7) = 0.97, *p* < 0.001), and for each partition individually (Spearman’s correlation (n = 168): r(166) = 0.90, *p* <0 .001). The high-throughput deep-learning pipeline provides validation to existing standards. This deep learning approach may benefit future studies in tracking baseline and resilient healthy aging to the earliest disease progression.

## Introduction

The hippocampus is a major hub of cognition and receives various inputs from the entorhinal cortex. It is crucial for cognitive processes like learning^1^, memory^2^, and spatial navigation^3^. Based on differences in location, cell morphology, and cytoarchitectural organization, the hippocampus can be parcellated into several subregions^4–8^. Subsequently, multiple studies emphasize the need for subregion-specific examinations, demonstrating subregional differences in functional specialization^9,10^, directly linking isolated subregion damages to distinct pathologies^11–13^, and pinpointing differences in vulnerability to neurodegeneration and cell death^13,14^. In neuroimaging, hippocampal volume is routinely used as a measure for disease progression. This is the case for neurodegenerative diseases such as Alzheimer’s disease^15,16^, as well as schizophrenia^17^. Reduced neuronal counts in the hippocampus have been linked to stress, depression, schizophrenia, and Alzheimer’s disease^18–20^. Stereology is a non-biased systematic random sampling method that has been used to produce total neuron counts in the hippocampal subregions and serves as a gold standard for neuron counting^18,21–23^. Two stereology methods find wide application, optical fractionator^24^ and the NvVref method^21,25^. Deep learning techniques have offered novel ways to quantify structure and have been successfully utilized in various fields, such as the diagnosis and classification of cancer^26,27^, the quantification of myofibers^28,29^, and the identification of histopathological markers in neurodegenerative diseases^30,31^. It has also been applied to segment fluorescently-tagged neurons in the human and rat brain^32,33^. Neuron segmentation in deep learning has been compared to stereology in previous studies; thus identifying it as a valuable and reliable method for the extraction of cell counts^34–38^. Yet it has not been applied to segment and quantify Nissl stained pyramidal neurons in the hippocampal subfields of the human brain—a region needed for cognition and severely affected in Alzheimer’s disease.

In this study, we created a pipeline for an automated (deep learning-based) extraction of pyramidal neuron estimates of the hippocampal subregions. Utilizing a unique dataset of histologically stained and parcellated hippocampal sections in the human brain, we piloted and applied a pre-trained convolutional neural network-based algorithm for cellular segmentation^39^ to segment neurons in the hippocampal pyramidal neuron layer. Moreover, we compared the deep learning generated pyramidal neuron segmentations with manual segmentations. We developed a filtering method ideal for hippocampal pyramidal neurons and compared our deep learning neuron estimates with manual stereology neuron counts using the optical fractionator probe and equation^24^. The goal of this study was twofold: (1) to develop a rigorously piloted pipeline to segment individual hippocampal neurons stained for Nissl, and (2) to extract the pyramidal neuron estimates of the human hippocampal subregions based on deep learning and relate to current standards in stereology. The aim was not to extract total neuron numbers of the entire hippocampus but to establish and present parameters for an automated high-throughput assessment of hippocampal pyramidal neuron numbers at the subregion level.

## Materials and methods

### Tissue samples

Seven human brain hemispheres (six left, one right) were acquired from the Massachusetts General Hospital Autopsy Suite (45–84 years; 68.33 ± 14.39 (mean ± sd); four males, two females, one unknown; postmortem intervals < 24 h). Autopsy consent was obtained from the legally empowered individual and autopsy tissue was collected only when allowance was made for research purposes. Excess tissue (defined as tissue not required for diagnostic purposes) was made available to investigators under a protocol approved by the IRB of MassGeneralBrigham. Samples were fixed by immersion in 10% formalin. Based on clinical reports, all cases were cognitive controls (cognitively healthy). All cases were screened for comorbidities based on the guidelines for the neuropathologic assessment of Alzheimer’s disease^40^ by the Massachusetts General Hospital Autopsy Suite. No cases with neurological, psychiatric, or infectious disease cases were included. All cases were assessed with gross tissue inspection and Luxol fast blue as well as Hematoxylin & Eosin staining were applied to rule out vascular disease and stroke. Immunohistochemistry for phosphorylated tau was used and all cases were staged for Braak & Braak (MPF, JCA)^41–43^. Subsequently, one case was diagnosed as normal control, three as Braak and Braak I, and three as Braak and Braak II. Table 1 lists relevant demographic information and Supplementary Table T1 lists the reagents used in this study.

**Table 1:** Basic demographic information for cases used in study.

Age	Hemisphere	Sex	PMI in hours	Braak & Braak stage	MTL amyloid burden	Cause of death	Clinical diagnosis
43	LH	F	24	NC	No	Ischemic renal injury	Cognitive control
59	LH	M	20	BBI	No	Liver failure	Cognitive control
68	RH	M	24	BBI	No	Acute cardiac death	Cognitive control
75	LH	M	24	BBII	Moderate	Vascular disease	Cognitive control
79	LH	M	15	BBI	High	Surgery complication	Cognitive control
84	LH	F	24	BBII	No	Pneumonia	Cognitive control
N/A	LH	N/A	24	BBII	No	N/A	Cognitive control

*BBI* Braak & Braak stage I, *BBII* Braak & Braak stage II, *cognitive control* cognitively healthy, *F* female, *M* male, *MTL* medial temporal lobe, *N/A* not available, *NC* normal control, *PMI* postmortem interval, *RH* right hemisphere, *LH* left hemisphere.

### Histology processing

Histology processing was based on a previous study^44^. First, tissue blocks were cryoprotected in 20% glycerol/2% dimethyl-sulfoxide-solution for a minimum of 10 days. Then, using a freezing sliding microtome (Leica Biosystems Inc, Buffalo Grove, IL USA), blocks were sectioned in the coronal plane at 50 µm and collected serially. The coronal plane is the accepted convention for hippocampal anatomy and reflects the orientation of hippocampal pyramidal layers. All sections were hand-mounted onto glass slides, dried overnight, and stained for Nissl substance with thionin. The staining protocol consisted of defatting (chloroform, 100% ethanol mixture, 1:1), pretreatment (acetic acid, acetone, 100% ethanol, double distilled water mixture, 1:1:1:1), staining in buffered thionin (8%), differentiating in 70% ethanol (addition of 5–10 drops of glacial acetic acid), dehydrating in an ethanol series (70%, 95%, 100%), clearing in xylene, and coverslipping with Permount.

### Subregion parcellation

**Figure 1:**
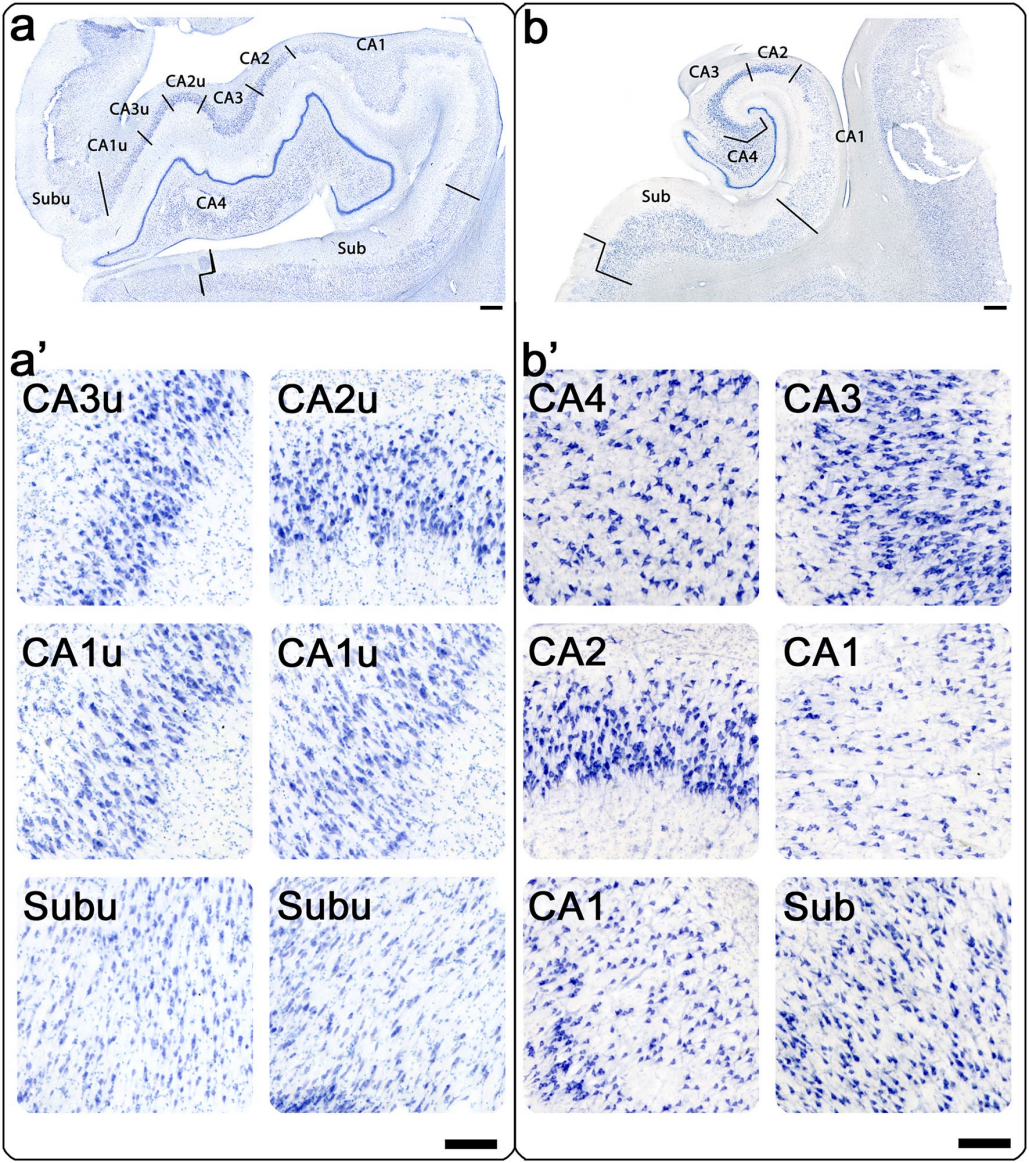
Cytoarchitecture characteristics of hippocampal subregions in Nissl staining: (**a**) medial (uncal) hippocampal subregions at the level of the hippocampal head: CA3u, CA2u, CA1u, Subu. (**a’**) Pyramidal layers of the medial hippocampal subregions. (**b**) Lateral hippocampal subregions at the level of the hippocampal body, clockwise: CA4, CA3, CA2, CA1, Sub (subiculum). (**b’**) Pyramidal layers of the lateral hippocampal subregions. Magnification bars in (**a, b**) = 1 mm; in (**a’,b’**) = 200 µm.

Our parcellation protocol was based on previous publications outlining distinct characteristics and appearances of the hippocampal subregions^4–8^. The hippocampus was parcellated into hippocampal subregions on each sampled slide (JCA, EMW). When the uncus is present, some hippocampal subregions occur medially as well as laterally. In our study, we refer to these additional medial subregions as CA1u (CA1 uncal), CA2u (CA2 uncal), CA3u (CA3 uncal), and Subu (Subiculum uncal), resembling the uncal regions defined by Ding and colleagues^5^. Each subregion was represented in the dataset (CA1, CA2, CA3, CA4, CA1u, CA2u, CA3u, Sub, and Subu), which consisted of a total of 168 partitions across subfields, cases, and levels. Figure 1a shows both the medial/uncal and lateral subregions at the level of the hippocampal head, Fig. 1b displays distinct characteristics of the lateral hippocampal subregion at the level of the hippocampal body.

### Tissue selection and digitization

The same tissue sections were selected and applied to both methods (deep learning and optical fractionator stereology) to quantify pyramidal neurons. The objective of this paper was not to quantify the total number of neurons in the hippocampus, but to test the deep learning pipeline by using a broad subset of hippocampal sections. Subsequently, stained hippocampal sections of each case were selected and sampled based on anatomical landmarks (five levels from anterior to posterior). The sampling levels were marked by millimeters from the first hippocampal slice and each level is approximate and rounded to the nearest whole number. The sampled levels were level 1, the anterior most sections of the hippocampus marked at 0 mm, level 2 is at 3.0 mm from the anterior most hippocampus, level 3 is located at 8.0 mm into the hippocampus, level 4 is at 11.0 mm, and lastly level 5 is at 15.0 mm. These hippocampal sampling levels (1. genu, 2. pes, 3. full dentate gyrus, 4. x-region, 5. body) have been further described with anatomical landmarks in a previous study (Williams et al. 2023)^8^. A total of 35 sections (seven cases x five coronal anterior–posterior levels) were sampled and digitized at high resolution (100× magnification) using a Keyence digital microscope (Keyence Corporation of America, Itasca, USA). The magnification is in line with several deep learning studies investigating histopathology on a cellular level^45–48^, offering sub-micrometer pixel sizes (0.75 µm × 0.75 µm), while limiting file volumes, and enabling time efficient processing.

### CellPose procedures (automated neuron estimates)

**Figure 2:**
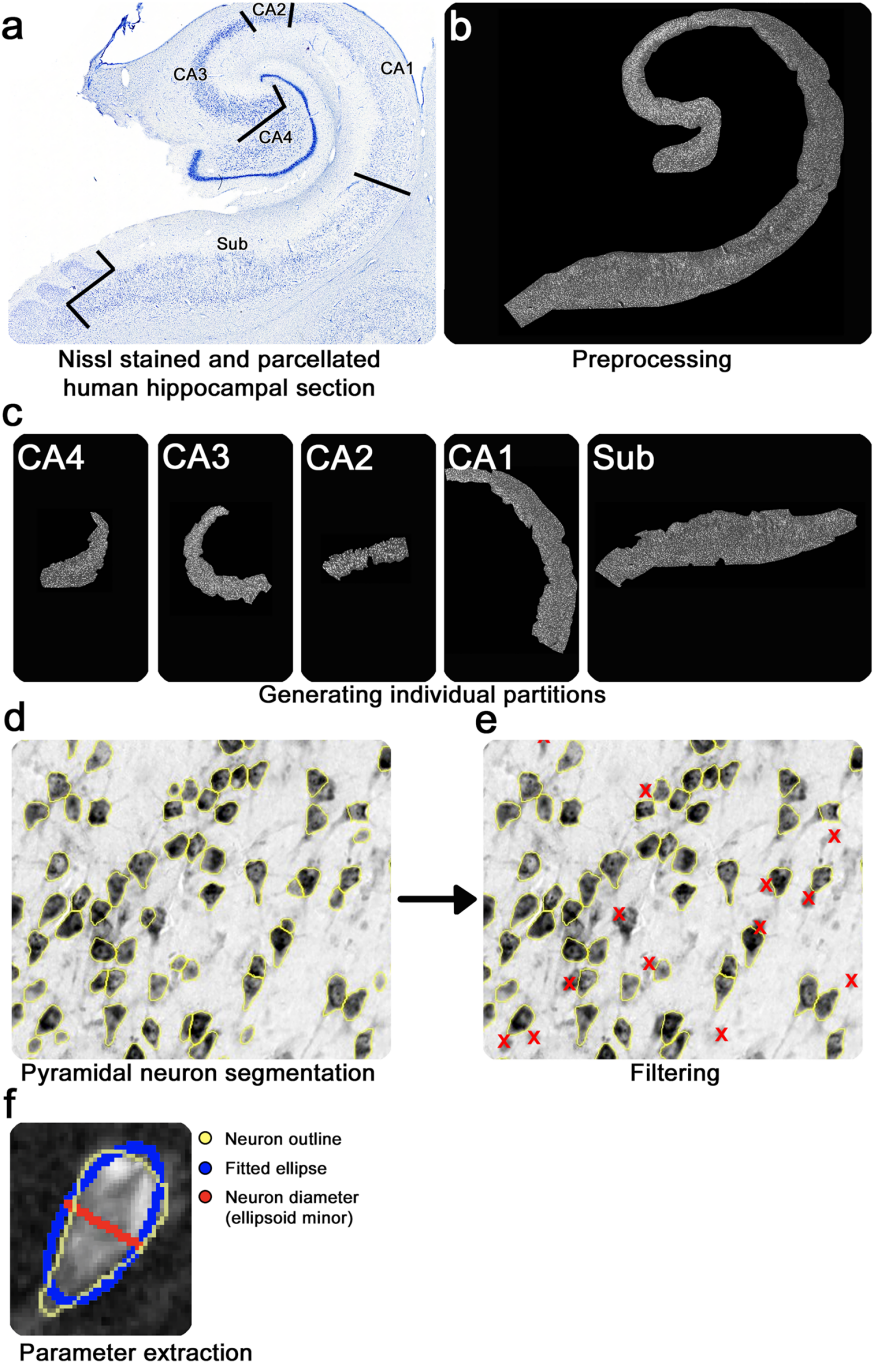
CellPose deep learning pipeline applied to hippocampal pyramidal neurons: (**a**) parcellated photomacrograph of hippocampus stained for Nissl substance (50 µm thick and coronal plane). (**b**) Cropped hippocampal pyramidal neuron layer to create partitions, transformed into inverted gray-value image. (**c**) Generating individual partitions. (**d**) Unfiltered segmented pyramidal neurons based on CellPose. Yellow outlines show neuron segmentations. (**e**) Filtered segmented neurons. Red X’s indicate removed items from segmentation (false-positives), (**f**) Hippocampal pyramidal neuron ellipsoid fitting and measurements (yellow: neuron segmentation, blue: fitted ellipse, red: neuron diameter (ellipsoid minor).

The deep learning extraction of subregion-specific pyramidal neuron estimates was conducted in four steps: (i) preprocessing, (ii) identifying deep learning parameters for hippocampal pyramidal neuron segmentation, (iii) establishing threshold-based filtering for false-positive segmentations, and (iv) extracting deep learning pyramidal neuron estimates based on CellPose. The deep learning pipeline utilized a high-performance cluster provided by the Massachusetts Life Sciences Center (MLSC). Figure 2 displays an overview of the complete pipeline for the CellPose-based extractions of pyramidal neuron estimates and related parameters.

#### (i) Preprocessing

Sampled slides were parcellated into subregions (Fig. 2a), and preprocessed to create optimal input slides^49^. The preprocessing consisted of transformation into an 8-bit image, cropping out the hippocampal pyramidal neuron layer, image inversion, and automated contrast and brightness adjustment (Fig. 2b**)**. Based on the cytoarchitectural parcellations of each sampled section, subregions were then manually separated and saved as individual partitions in the .tiff format (Fig. 2c). Each step was conducted using Fiji/ImageJ v1.53 (https://www.imagej.nih.gov).

#### (ii) Determining deep learning parameters for hippocampal pyramidal neuron segmentation

Our study employed the neural network-based algorithm CellPose v0.6^39^. CellPose is a generalist algorithm for cellular segmentation, which has been pre-trained and evaluated to accurately segment a diverse set of cellular shapes and sizes^28,39,49^. CellPose has been previously applied for detailed histopathological analyses like the automated and unbiased quantification of highly uneven myofibers^28^. To ensure optimal segmentation for our dataset of hippocampal pyramidal neurons, thorough piloting of algorithmic input parameters was conducted. To this aim, a subset of 18 randomly selected partitions (three cases, each subregion represented twice) was processed using five different size input parameters (i.e., flexible pixel diameter, 23 pixel diameter, 24 pixel diameter, 26 pixel diameter, or 27 pixel diameter). The pixel diameter parameter was used to specify the approximate diameter of the cells in pixel that will be segmented. It is used to scale the convolutional filters applied to the image, which affects the size of the objects that the algorithm is able to detect^39^. The resulting segmentations per size input parameter were then visually inspected (JCA, EMW), and ranked for most accurate segmentations^39^. Supplementary Fig. F1 displays the piloting of CellPose size input parameters (i.e., flexible diameter, 23 pixel diameter, 24 pixel diameter, 26 pixel diameter, or 27 pixel diameter). Based on our piloting, the best-performing size input parameter for hippocampal pyramidal neurons was identified (24 pixel diameter), and employed for the processing of all individual partitions (n = 168). It reliably segmented pyramidal neurons of each subregion, minimizing false-negatives and false-positive segmentations, as well as over- and under-segmentation. Figure 2d shows an example vignette of the resulting segmented pyramidal neurons. Segmentations were visualized using Fiji/ImageJ v1.53. For a complete list of the CellPose input parameters and model used for the pipeline, see Supplementary Table T2.

#### (iii) Establishing threshold-based filtering parameters of segmented neurons

Based on the 168 processed partitions, we encountered four possible instances of false-positive segmentations of pyramidal neurons: (1) segmented extracellular space, (2) glial cells, (3) neuron profiles (partial neurons), and (4) overlapping pyramidal neurons (combined pyramidal neurons). The term neuron profile refers to a partial pyramidal neuron that was not parallel to the coronal plane or only partially displayed. Similar to glial cells, neuron profiles were smaller than in plane pyramidal neurons. Supplementary Fig. F2 shows a segmented pyramidal neuron and the four instances of false-positive segmentations. To correct each possible instance of false-positive segmentations, we developed an automated filtering method based on piloting and evaluation.

To exclude segmentations that were extracellular space, the mean gray value of the partition was set as a threshold (extracellular space and neurons). Segmentations lighter than this threshold were likely not pyramidal neurons and removed. To remove glial cells and neuron profiles, each segmentation was ellipsoid fitted (Fig. 2f**,** blue circle)^50^. Based on the geometry of pyramidal neurons, the minor axis of the ellipse (width) resembles the pyramidal neuron diameter (Fig. 2f**,** red line). Subsequently, a lower threshold for pyramidal neuron diameter for each partition was calculated and segmentations under this threshold were excluded. To identify an ideal filtering threshold a subset of 18 randomly selected partitions (three cases, each subregion represented twice) was filtered using a set of lower diameter thresholds (mean—1SD; mean—0.75SD, mean—0.5SD) and inspected for accuracy by JCA and EMW. Filtered segmentations were then ranked for best filtering performance based on the exclusion of the aforementioned false-positive segmentations, whilst correctly segmented and delineated pyramidal neurons remained untouched. Thus, optimal filtering parameters were identified (mean diameter—0.75SD) and applied to each partition (n = 168). We did not implement a similarly piloted upper threshold that would exclude overlapping neurons. This allowed us to count overlapping neurons as one, while excluding neuron profiles and glial cells. Supplementary Fig. F3 displays the piloting of different filtering parameters. Figure 2d displays the unfiltered pyramidal neuron segmentations generated by CellPose, and Fig. 2e shows segmented hippocampal pyramidal neurons post-filtering. Filtering was conducted using Fiji/ImageJ v1.53 and each individual neuron was numbered and trackable throughout the filtering and analyses process.

#### (iv) Extracting pyramidal neuron estimates

Pyramidal neuron estimates of each of the 168 partitions were extracted, equaling the number of segmented pyramidal neurons post-filtering. Per partition, filtered and unfiltered pyramidal neurons were assigned a number and partitions tallied into .tiff and .csv files to enable trackability.

### Assessment of segmentations (masks) generated by deep learning

To test the filtered pyramidal neuron segmentations generated by the CellPose deep learning pipeline^39^, we directly compared them to manual segmentations. This approach was based on a similar paper applying CellPose for the automated unbiased segmentation and quantification of myofibers^28^. Five vignettes containing a minimum of 75 pyramidal neurons were created from randomly selected partitions (750 µm × 750 µm, five cases, no subregion represented twice). Pyramidal neurons within these vignettes were then automatically segmented using the deep learning pipeline, as well as manually by three raters respectively (JR, EWR, JO). Manual segmentation was based on the filtering and segmentation criteria reported above, avoiding overfitting and underfitting of segmentations, and excluding the following: extracellular space, glial cells, neuron profiles, and overlapping neurons. In both methods pyramidal neurons touching the edge of a vignette were excluded. The creation of manual masks was conducted using Fiji/ImageJ v1.53. Dice scores of automated versus manual pyramidal neuron segmentations (masks) in five individual vignettes were calculated based on pixel wise comparison of the binarized masks using the MorphoLibJ plugin for ImageJ/Fiji^51^.

### Stereology (optical fractionator, manual neuron counts)

**Figure 3:**
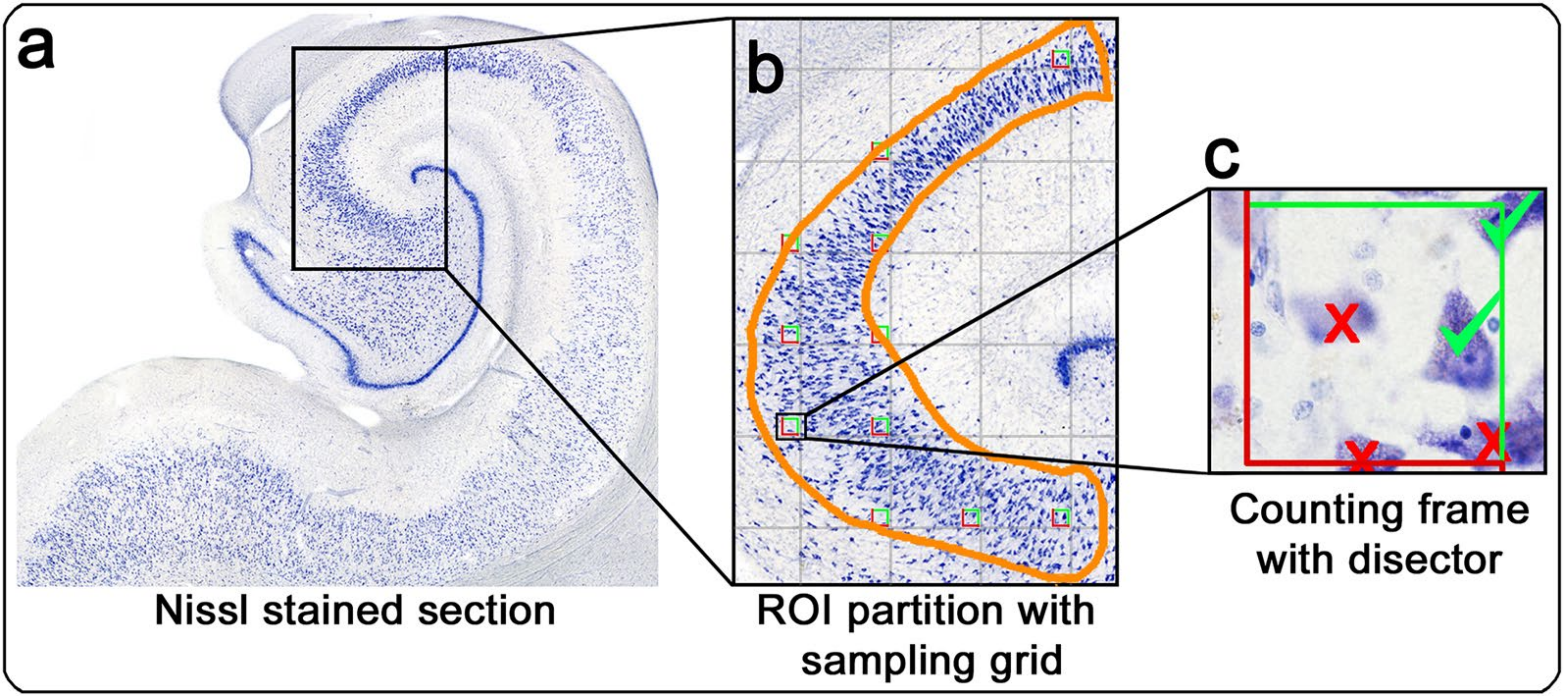
Manual hippocampal pyramidal neuron counts using optical fractionator stereology and systematic random sampling: (**a**) Nissl stained section with inset showing CA3 (example ROI). (**b**) ROI was outlined with random sampling grid overlaid and resulting with approximately 10 counting frames per the ROI. (**c**) 50 µm × 50 µm counting frame showing exclusion (red) and inclusion (green) lines. Tissue thickness taken by focusing from top to bottom of the section and averaged 20.3 µm across partitions. *ROI* region of interest.

To obtain neuron counts for validation, we applied a systematic random sampling protocol for manual stereology, utilizing the optical fractionator^24^. Manual stereology counts for validation were conducted by EWR using Stereoinvestigator v2021.1.3 (MicroBrightField Inc, Burlington VT), a Nikon80i microscope attached to a Ludl motorized stage, and the optical fractionator probe. Each partition was traced as an ROI (region of interest) with the 4× objective. We used a 100× oil objective and an average of 10 counting frames per subregion with a disector height of 10 μm. Counting frame size was 50 μm × 50 μm. Two guard zones, 3 μm each, were included at the top and bottom of the counting frame. Counting criteria included a neuron with nucleolus, which was in focus within the disector, and does not cross the red exclusion lines of the counting frame. Section thickness was measured at each counting frame (mean tissue thickness across partitions = 20.39 µm). The number of neurons per partition (N) was calculated by the Stereoinvestigator software utilizing the optical fractionator equation provided by West and Gundersen^24^: $N ={\sum }Q * \frac{t}{h}*\frac{1}{asf}*\frac{1}{ssf}$. The optical fractionator equation takes into account the following parameters: Q = number of neurons counted (varied per counting frame), t = tissue thickness of the section, h = height of the counting frame, asf = area sampling fraction, and ssf = section sampling fraction. Since the aim of this approach was not to extract total neuron numbers of the hippocampus, but to test deep learning estimates of 168 individual hippocampal partitions against manual stereology neuron counts of the same partitions, the coefficient of Error (CE) was not applicable and ssf set to 1. The stereology pipeline for manual pyramidal neuron counts is illustrated in Fig. 3.

### Development and availability of deep learning pipeline

Each in-house developed script was built as a macro/plugin for Fiji/ImageJ v1.53, and has been made available on GITHUB (https://github.com/AugustinackLab/NeuronNumberEstimates). The set of scripts include: preprocessing, automated filtering of false-positive segmentations, and parameter extraction as well as subsequent preparation of results for analysis and quality control. To facilitate application, scripts were annotated and input parameters such as filtering threshold can be modified. Standards and parameters required for digitization were included.

### Statistical analysis

Statistical analysis and data presentation were conducted using Prism v.9.1 (Graphpad, https://www.graphpad.com) and R-Studio v.1.4.1 (The RStudio Team, https://www.r-project.org). Multiple Shapiro–Wilk-tests were computed to screen for violations of normality. First, differences between Dice scores of automated versus manual pyramidal neuron segmentations and pairs of manual segmentations were quantified using an Independent Samples *t-*Test. Second, we used a Paired Samples Wilcoxon Test to compare automated CellPose pyramidal neuron estimates and manual stereology counts of each partition. Third, two Kruskal–Wallis-H tests were conducted to investigate subregion differences, using either manual neuron counts or automated neuron estimates per partition as independent variables, and subregion (CA1, CA1u, CA2, CA2u, CA3, CA3u, CA4, Sub, Subu) as a factor. Fourth, a Spearman's correlation was computed to reveal correlations in automated pyramidal neuron estimates and manual pyramidal neuron counts per subregion. Fifth, a Spearman’s correlation was used to reveal associations of automated pyramidal neuron estimates and manual pyramidal neuron counts of individual partitions. Post-hoc testing was conducted with Dunn’s tests in combination with Holm's correction for multiple comparisons. All statistical tests were two-sided and utilized *p* < 0.05 as the level of significance.

## Results

### Deep learning segments pyramidal neurons

For automated pyramidal neuron estimates, we show the unfiltered (Fig. 2d) as well as the finalized threshold filtered segmentations (Fig. 2e). The unfiltered segmentation of hippocampal pyramidal neurons across all cases, levels, and subregions totaled 631,494. Post filtering, 479,873 pyramidal neurons remained (75.99% ± 0.26; mean ± S.E.M.). Approximately a quarter of the initially segmented neurons across partitions was therefore excluded (24.01% ± 0.26; mean ± S.E.M.). Supplementary Table T2 lists percentages of pyramidal layer neuron segmentations remaining post-filtering averaged per subregion. Supplementary Table T4 provides subregion specific descriptive statistics of unfiltered pyramidal layer neuron estimates.

### Assessment of neuronal segmentation

**Figure 4:**
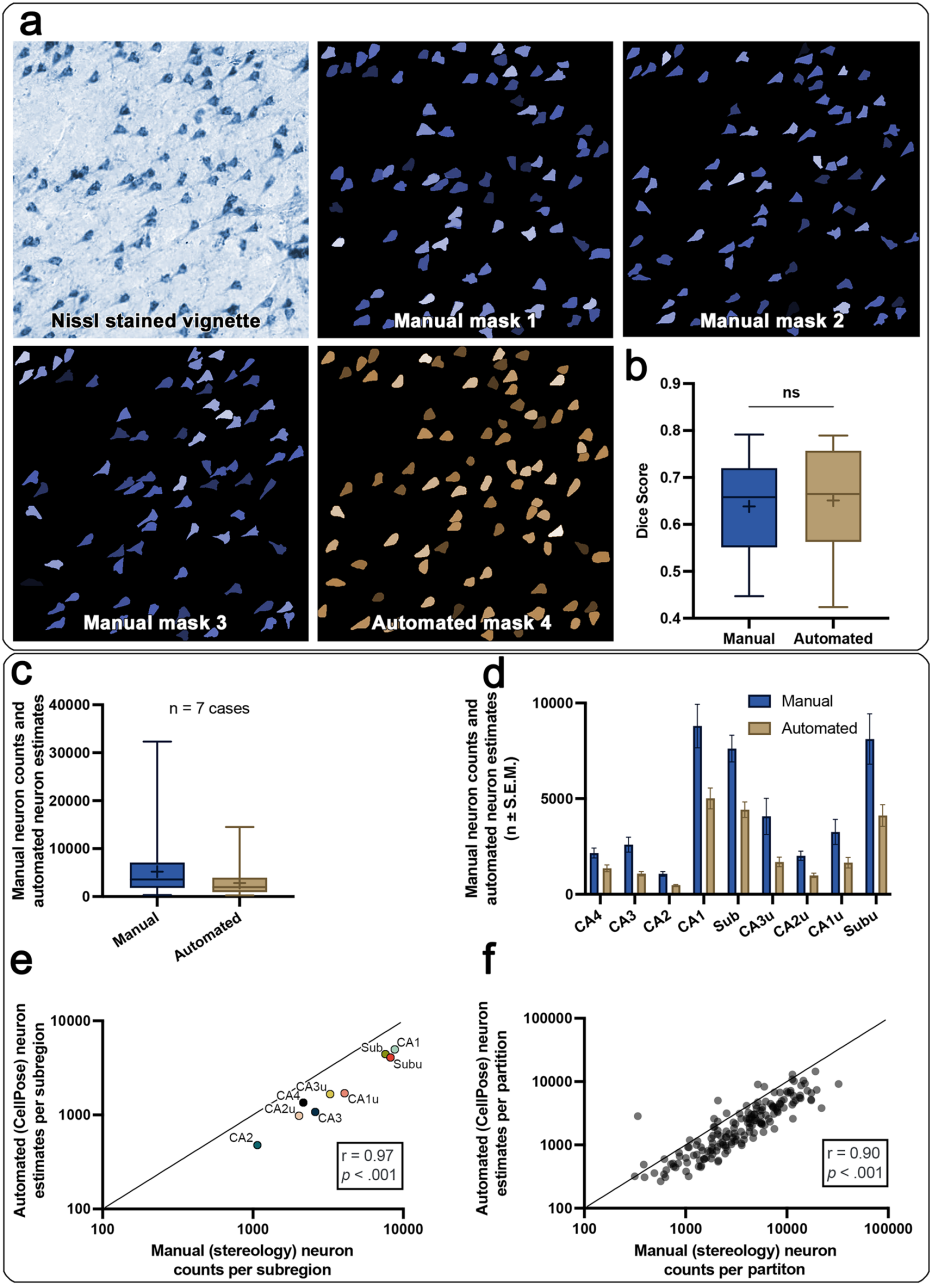
Correlation of the CellPose deep learning pipeline: (**a**) Nissl stained vignette used for pyramidal neuron segmentation, three raters performed manual segmentations (masks 1, 2, 3; blue), and CellPose performed the automated segmentation (mask 4; brown). (**b**) No significant difference between the Dice scores calculated from the manual masks (manual rater versus manual rater), and automated masks (automated versus manual rater). Whiskers indicate min to max, + indicates mean, line indicates median. (**c**) Manual pyramidal neuron counts and automated pyramidal neuron estimates. Averaged across subregions, levels and cases. Whiskers indicate min to max, + indicates mean, line indicates median. (**d**) Subregion specific pyramidal layer neuron estimates, averaged across cases and levels. (**e**) Excellent correlation of manual (stereology) and automated (CellPose) pyramidal layer neuron estimates averaged per subregion (Spearman’s correlation (n = 9): r(7) = 0.97, *p* < 0.001). (**f**) Excellent correlation of manual (stereology) and automated (CellPose) pyramidal layer neuron estimates per partition (not averaged per subregion; Spearman’s correlation (n = 168): r(166) = 0.90, *p* < 0.001).

To assess the automated pyramidal neuron masks created by the deep learning pipeline, we compared them with manual pyramidal neuron segmentations (three raters: JR, EWR, JO). Dice scores were utilized to quantitatively investigate the similarity of CellPose masks with manual segmentations (automated versus rater), as well as manual segmentations with manual segmentations (rater versus rater). The mean Dice score of automated masks versus manually created segmentations was 0.65 ± 0.03 (mean ± S.E.M.), while the mean Dice score of pairs of manually created segmentations was 0.64 ± 0.03 (mean ± S.E.M.). Figure 4a shows the automated CellPose masks, as well as manually segmented pyramidal neurons of the same vignettes. There was no significant difference between Dice scores of automated versus rater, and rater versus rater segmentations (paired samples *t* test: *t*(28) = 0.33, *p* = 0.742, Fig. 4b). Supplementary Table T4 lists additional automated and manual comparisons.

### Manual pyramidal neuron counts correlate with deep learning estimates

The same cases, levels, and hippocampal subregions were represented in automated CellPose neuron estimates and manual stereology neuron counts. A Paired Samples Wilcoxon Test showed significantly higher manual (stereology) pyramidal neuron counts than automated (CellPose) pyramidal neuron estimates (n = 168, W = −12,832, *p* < 0.001). The average pyramidal neuron count across subregions was 2815.74 ± 204.31 (median = 1980.00) in automated estimates and 5184.37 ± 382.07 (median = 3565.35) in manual counts (both mean ± S.E.M.) (Fig. 4c). The automated neuron estimates across subregions, levels, and cases totaled to 470,873, compared to 870,974 in manual counts. As the numbers demonstrate, automated estimates were routinely lower than manual counts. However, both methods show a strong and consistent relationship across subregions, as displayed in Fig. 4d. A Spearman’s correlation compared automated neuron estimates and manual neuron counts averaged per subregion, and revealed an excellent positive association (Spearman’s correlation: n = 9, r(7) = 0.97, *p* < 0.001) (Fig. 4e). Similarly, we observed an excellent correlation between automated neuron estimates and manual neuron counts of individual partitions (Spearman’s correlation: n = 168, r(166) = 0.90, *p* < 0.001) (Fig. 4f). Two Kruskal–Wallis H-tests revealed a significant effect of subregion in automated neuron estimates as well as manual neuron counts (automated: n = 168, *χ*^*2*^(8) = 121.4, *p* < 0.001; manual: n = 168, *χ*^*2*^(8) = 96.65, *p* < 0.001). Automated pyramidal neuron estimates showed consistently lower variability in comparison to the manual method (Fig. 4d). Table 2 shows descriptive statistics of automated neuron estimates and manual neuron counts per subregion. Furthermore, post-hoc comparisons showed the same pattern of subregions differing significantly from each other in manual stereology and the CellPose deep learning pipeline (Table 3).

**Table 2:** Descriptive statistics of hippocampal subregion CellPose pyramidal neuron estimates and manual neuron counts.

Variable of interest	Sub-region	n datap	25% Perc	Median	75% Perc	Mean	Std. dev	S.E.M	Lower 95% CI	Upper 95% CI
Pyramidal neuron estimates, automated (CellPose)	CA1	35	2441	4424	6625	5014	3246.40	548.74	3898.80	6129.20
	CA1u	9	1015	1382	2386	1652.70	830.28	276.76	1014.50	2290.90
	CA2	21	332.50	455	583.50	478.19	152.29	33.23	408.87	547.51
	CA2u	11	785	933	1148	990.27	366.81	110.60	743.84	1236.70
	CA3	20	645	997	1263	1075.80	518.25	115.89	833.20	1318.30
	CA3u	10	1173.30	1755	2361.30	1692.90	787.16	248.92	1129.80	2256
	CA4	15	917	999	1338	1269.50	635.82	164.17	917.43	1621.60
	Sub	35	2918	3655	5592	4423.10	2387.50	403.55	3603	5243.30
	Subu	12	2136	4407.50	5362.30	4120.70	1971.10	569.01	2868.30	5373.10
Pyramidal neuron counts, manual (stereology)	CA1	35	3537	7173.70	13,529	8798.90	6729.30	1137.50	6487.30	11,110
	CA1u	9	2073.90	2355.30	5211.50	3257	1968.90	656.31	1743.60	4770.50
	CA2	21	622.26	861.08	1472.20	1068.40	573.47	125.14	807.36	1329.40
	CA2u	11	1513.10	1794.80	2838.20	2015	801.69	241.72	1476.50	2553.60
	CA3	20	1083.30	2478.40	3625.40	2594.60	1741	389.30	1779.80	3409.40
	CA3u	10	1759	3861	4648.90	4072.70	2974.10	940.51	1945.10	6200.30
	CA4	15	1421.30	2203.40	2520.30	2159	994.10	256.68	1608.50	2709.50
	Sub	35	4954.60	6039	10,225.0	7619.40	4130.90	698.26	6200.40	9038.40
	Subu	12	4467.40	7534.60	12,060	8118.10	4562.90	1317.20	5218.90	11,017

25% percentile, median, 75% percentile, mean, std. dev., S.E.M., lower 95% confidence interval and upper 95% confidence interval. In both methods CA1 displays the largest variability and neuron estimates.

**Table 3:** Direct comparison of hippocampal subregion pyramidal layer neuron counts.

*Variable of interest*	Compared subregions	n datapoints	Manual (stereology)			Automated (CellPose)		
			Z	p adj	signif	Z	p adj	signif
Manual pyramidal neuron counts (stereology), automated pyramidal neuron estimates (CellPose)	CA1 vs. CA1u	35 vs. 9	2.50	0.444	ns	3.03	0.09	ns
	CA1 vs. CA2	35 vs. 21	**7.13**	** < 0.001**	****	**8.26**	** < 0.001**	****
	CA1 vs. CA2u	35 vs. 11	**4.18**	** < 0.001**	**	**4.72**	** < 0.001**	****
	CA1 vs. CA3	35 vs. 20	**4.41**	** < 0.001**	***	**5.64**	** < 0.001**	****
	CA1 vs. CA3u	35 vs. 10	2.16	> 0.999	ns	3.14	0.06	ns
	CA1 vs. CA4	35 vs. 15	**4.39**	** < 0.001**	***	**4.54**	** < 0.001**	***
	CA1 vs. Sub	35 vs. 35	0.32	> 0.999	ns	0.16	> 0.999	ns
	CA1 vs. SubU	35 vs. 12	0.17	> 0.999	ns	0.32	> 0.999	ns
	CA1u vs. CA2	9 vs. 21	2.59	0.346	ns	2.89	0.141	ns
	CA1u vs. CA2u	9 vs. 11	1.13	> 0.999	ns	1.12	> 0.999	ns
	CA1u vs. CA3	9 vs. 20	0.75	> 0.999	ns	1.12	> 0.999	ns
	CA1u vs. CA3u	9 vs. 10	0.35	> 0.999	ns	0.01	> 0.999	ns
	CA1u vs. CA4	9 vs. 15	1.00	> 0.999	ns	0.64	> 0.999	ns
	CA1u vs. Sub	9 vs. 35	2.71	0.246	ns	2.92	0.124	ns
	CA1u vs. SubU	9 vs. 12	2.25	0.881	ns	2.32	0.736	ns
	CA2 vs. CA2u	21 vs. 11	1.40	> 0.999	ns	1.74	> 0.999	ns
	CA2 vs. CA3	21 vs. 20	2.34	0.701	ns	2.24	0.915	ns
	CA2 vs. CA3u	21 vs. 10	3.11	0.068	ns	3.00	0.097	ns
	CA2 vs. CA4	21 vs. 15	1.81	> 0.999	ns	2.6	0.332	ns
	CA2 vs. Sub	21 vs. 35	**7.40**	** < 0.001**	****	**8.13**	** < 0.001**	****
	CA2 vs. SubU	21 vs. 12	**5.59**	** < 0.001**	****	**6.00**	** < 0.001**	****
	CA2u vs. CA3	11 vs. 20	0.55	> 0.999	ns	0.13	> 0.999	ns
	CA2u vs. CA3u	11 vs. 10	1.54	> 0.999	ns	1.16	> 0.999	ns
	CA2u vs. CA4	11 vs. 15	0.22	> 0.999	ns	0.59	> 0.999	ns
	CA2u vs. Sub	11 vs. 35	**4.40**	** < 0.001**	***	**4.61**	** < 0.001**	***
	CA2u vs. SubU	11 vs. 12	**3.60**	**0.012**	*	**3.65**	**0.009**	**
	CA3 vs. CA3u	20 vs. 10	1.20	> 0.999	ns	1.17	> 0.999	ns
	CA3 vs. CA4	20 vs. 15	0.35	> 0.999	ns	0.53	> 0.999	ns
	CA3 vs. Sub	20 vs. 35	**4.68**	** < 0.001**	***	**5.51**	** < 0.001**	****
	CA3 vs. SubU	20 vs. 12	**3.54**	**0.014**	*	**4.04**	**0.002**	**
	CA3u vs. CA4	10 vs. 15	1.43	> 0.999	ns	0.67	> 0.999	ns
	CA3u vs. Sub	10 vs. 35	2.37	0.643	ns	3.04	0.086	ns
	CA3u vs. SubU	10 vs. 12	1.94	> 0.999	ns	2.38	0.625	ns
	CA4 vs. Sub	15 vs. 35	**4.64**	** < 0.001**	***	**4.42**	** < 0.001**	***
	CA4 vs. SubU	15 vs. 12	**3.65**	**0.010**	**	**3.34**	**0.031**	*
	Sub vs. SubU	35 vs. 12	0.06	> 0.999	ns	0.21	> 0.999	ns

Compared subregions, number of subregions (n datapoints), as well as for both methods (automated CellPose versus manual stereology) q-statistic, adjusted p-value and significance. Computed using Kruskal–Wallis H-tests and corrected for multiple comparisons using Dunn’s tests. Significant differences are shown in bold. *p < 0.05, **p < 0.01, ***p < 0.001, ****p < 0.0001.

### Time allotment comparison

The automated deep learning pipeline used 27 h of manual preparation. This preparation time included writing scripts (20 h), determining optimal segmentation parameters (two hours), piloting optimal filtering parameters for the removal of false-positive segmentations (three hours), and preprocessing the input slides (cropping out of pyramidal layer and generation of individual partitions; two hours). In addition, the deep learning pipeline utilized seven hours of computing time on a high-performance cluster. By comparison, the manual stereology neuron counts on the same partitions required 35 h of labor by stereology counter (EWR). This time allowance includes set up times for the experiments. The histological sections processed for our study were already digitized (approximately 5–10 sections per hour). Both methods utilized the same tissue and parcellations. Thus, tissue processing as well as parcellation times were not included in time estimates.

## Discussion

In this study, we present a deep learning-based pipeline to extract subregion-specific neuron estimates of the hippocampal pyramidal layer and relate our results to the current standards for stereology. We used the pre-trained and successfully applied convolutional neural network algorithm for cellular segmentation “CellPose”^28,39^ to delineate pyramidal neurons in 168 hippocampal samples, and developed an automated filtering method for false-positive segmentations. This report establishes tailored input and filtering parameters. It provides a pipeline for high-throughput pyramidal neuron quantifications in the human hippocampal subregions that can be expanded to additional datasets. To the best of our knowledge, it is the first to show that deep learning-based estimates approximate the relative ratio of pyramidal neurons in manual stereology counts of the human hippocampal subregions. Our results show no difference in Dice scores of pyramidal neurons created by the CellPose pipeline and segmentations conducted by manual raters. While both methods resulted in different total pyramidal neuron counts, our data demonstrates excellent correlations of pyramidal neuron estimates extracted using the deep learning approach with manual stereology experiments. Notably, automated pyramidal neuron estimates consistently revealed lower variability. This work establishes parameters for the CellPose deep learning pipeline in the hippocampal pyramidal neurons and provides needed scripts, which future studies can utilize. The difference between time allotments suggests that the deep learning approach is time efficient, while requiring minimal human intervention.

There was no significant difference when comparing Dice scores of automated versus manual segmentations and pairs of manual segmentations (*t*(28) = 0.33, *p* = 0.742). This finding is in line with a recent study which applied the same convolutional neural network (CellPose) to segment and quantify myofibers^28^. Moreso, we observed significant and exceptionally strong associations between manual and automated pyramidal neuron quantifications. This was true per subregion (Fig. 4e), as well as per individual partition (Fig. 4f). Although we observed significant differences in the raw numbers between methods (Fig. 4d), automated neuron estimates adhered to the same pattern as manual neuron counts (Fig. 4c), revealing the same pattern among subregions. Automated neuron estimates and manual neuron counts employed vastly different approaches. Yet, the same relationship was observed, and direct comparisons resulted in the same differences among subregions across methods (e.g., CA1 and CA2 significantly different in both methods).

Manual neuron counts were conducted using stereology and based on the well-established optical fractionator method^24^. The optical fractionator method employs systematic random sampling as well as other guiding parameters to avoid bias. It incorporates a disector, guard zones as well as counting rules to avoid neuron profiles^21,24,52^. Yet, it is labor-intensive, and relies completely on human intervention, which can introduce subjectivity. Subjectivity, differences in training, recognition biases, and fatigue might potentially influence results^34,35^. Deep learning has been applied in various previous works in humans and animal models^26,27,30,31,53^. It was identified as an effective and reliable method for cell counting by negating interrater variability and enabling reproducibility^34,35^. The numbers generated with traditional stereology represent a small percentage of the total population. Yet they have human evaluation and attention which may pose a strength and a weakness. This deep learning method reduces human intervention to the initial delineation of subregions. It labels most neurons within the partition, implements strict filtering of false-positive segmentations (75.99% of neuron segmentations remained post-filtering), and provides a quantitative number for hippocampal pyramidal neuron counts. Of course, no method is perfect. Yet the test of the two methods suggests the CellPose deep learning approach is on par with stereology when identifying relative differences in hippocampal subregions based on neuron counts. This study’s pipeline reduces labor, enables a fast assessment of larger datasets, and potentially minimizes biases due to human factors.

Previous works quantifying neuron counts in the human hippocampus usually did not assess the uncal subregions, or differentiate CA2 and CA3^18,21–23,54^. Our study reports deep learning-based neuron estimates for the lateral and medial subregions of the human hippocampus. Also, it analyzes CA2 and CA3 separately and individually. Both methods (deep learning and manual stereology) resulted in CA1, Sub, and Subu as having the largest number of hippocampal pyramidal neurons, while CA2 and CA2u had the lowest. Given that CA1, Sub, and Subu were the largest subregions, while the size of CA2 and CA2u was small, the outcome of neuronal number is fitting. Our observed pattern of pyramidal neuron estimates between subregions is in line with previous works on older normal controls and preclinical Alzheimer's disease patients^18,21–23,54^. For example, West and Gundersen (1990) and Simić (1997) reported that CA1 contained the most neurons, followed by subiculum, and CA3 and CA2 together (Supplementary Table T5). Our study provides correlated neuron estimates using deep learning, establishing a measure in the human hippocampus utilizing a novel approach. Though it does not report total neuron counts of the human hippocampus (more sections would be needed), it demonstrates the same pattern of neurons (manual counts versus CellPose estimates) between respective subregions. Our work serves as a correlation study for the deep learning approach for the human hippocampus.

Estimating neuron counts in the human brain using a high yield method is an extremely attractive method to better understand neuronal death in aging and disease. That is, neuronal death may occur with a pathological marker (neurofibrillary tangle), or without one. Tau severity has been well documented in numerous staging studies^41,42,55–59^. Studies documenting neuronal loss in Alzheimer’s disease on the other hand have been far less common^25,60,61^. This can be attributed to the fact that stereology has numerous requirements to achieve unbiased protocol. Using high-throughput deep learning methods may allow for a better understanding of the relationship among neurofibrillary tau tangles, amyloid-beta, and neuronal death. Future work will have to further investigate this.

This study has some limitations and technical notes. First, the CellPose algorithm^39^ was successfully applied in previous studies^28,29^, yet it yielded some false-positive segmentations. This limitation was however minimized by vetting ideal input parameters for segmentation and automated filtering for false-positive segmentations. Second, the CellPose algorithm in some instances did not segment pyramidal neurons in highly dense areas. Given that hippocampal subregions differ in pyramidal neuron density, the CellPose deep learning method may undercount in high density subregions such as CA3. Third, the deep learning method works in 2D and counts overlapping neurons as one neuron, rather than the actual number in the neuron grouping. Clustered neurons being counted as one was implemented to remove glial cells and neuronal profiles from the segmentations, which were generally smaller than pyramidal neurons. On the other hand, stereology utilizes the ability to assess multiple focal planes; it counts in 3D and overlapping cells were not an exclusion criterion. Future approaches might investigate the application of deep learning to quantify pyramidal neurons in 3D. Fourth, our study adhered to common practice and assessed the hippocampus at the coronal plane, which is in plane with the orientation of human hippocampal pyramidal neurons^7,8,62–64^. A small fraction of neurons may be oriented nonparallel to the plane and might consequently be excluded as neuron profiles. This is minimized in the coronal plane. Automated neuron estimates and manual neuron counts employed vastly different approaches, nonetheless, the same relationship between subfields was observed. Taken together, the aforementioned limitations might be a reason for the difference in pyramidal neuron numbers between the two methods. The quality of staining and digitization of histologic sections varies due to technological factors^30,34,35^. Convolutional neural networks like CellPose are however based on the detection of patterns and shapes from image data. Thus, they do not rely on predefined ranges of pixel color and intensity, nor on human interaction^65^, and potentially add a level of resilience against biasing factors reported above.

Our study provides a pipeline for automated pyramidal neuron quantifications of the human hippocampal subregions. While the CellPose deep learning pipeline does not generate the same total numbers, it shows the same relative ratios as performed by manual stereology. The deep learning pipeline is made available as open-source. Parameters were rigorously piloted and are applicable to future works quantifying pyramidal neurons in the hippocampal subregions. Thus, this study may enable the analysis of much larger datasets and potentially facilitate the harmonization of histopathological analysis across multiple cohorts. This pipeline has the ability to crunch and produce “big data” numbers, which is an attractive approach for the investigation of neuronal death and its relation to tau pathology. Our work creates a baseline for more specificity in tracking resilient healthy aging in the human hippocampus. Investigating subregion-specific vulnerability to neuronal loss will not only benefit early diagnosis, but also help understand pathological mechanisms and progression of neurodegenerative diseases on a cellular level.

## Supplementary Information

Supplementary Information.

## Data Availability

The datasets generated during and/or analyzed during the current study are available from the corresponding author on reasonable request. The scripts generated for this study are available on GITHUB (https://github.com/AugustinackLab/NeuronNumberEstimates).

## Abbreviations

BB: Braak & Braak stage
CA1: Cornu ammonis 1
CA2: Cornu ammonis 2
CA3: Cornu ammonis 3
CA4: Cornu ammonis 4
Sub: Subiculum
CA1u: CA1 uncal
CA2u: CA2 uncal
CA3u: CA3 uncal
