# Supplementary material for: Stereology neuron counts correlate with deep learning estimates in the human hippocampal subregions
Source: Sci Rep. 2023 Apr 11;13:5884. doi: 10.1038/s41598-023-32903-y (PMC10090178; doi:10.1038/s41598-023-32903-y)
Supplement: Supplementary file 1 — Supplementary Information. [file 41598_2023_32903_MOESM1_ESM.pdf]

Stereology neuron counts validate deep learning estimates in the human hippocampal subregions

Oltmer, J[1,2], Rosenblum, EW[1], Williams, EM[1], Roy, J[1], Llamas-Rodriguez J[1], Perosa, V[3,4], Champion, SN[5], Frosch, MP[5], Augustinack, JC[1,2*]

Author affiliations:

1 Athinoula A. Martinos Center, Massachusetts General Hospital, Department of Radiology, Charlestown, MA, USA

2 Harvard Medical School, Boston, MA, USA

3 Department of Neurology, Massachusetts General Hospital, Harvard Medical School, J. Philip Kistler Stroke Research Center, Boston, MA, 02114, USA

4 Department of Neurology, Otto-Von-Guericke University, Magdeburg, Germany

5 Department of Neuropathology, Massachusetts General Hospital, Boston, MA, USA

Corresponding author *:

Jean C Augustinack

Full address: Department of Radiology, Athinoula A. Martinos Center for Biomedical Imaging, Massachusetts General Hospital, Building 149 –13th St. Room 2301, Charlestown, MA, 02129, USA. E-mail: jaugustinack@mgh.harvard.edu

Reagent	*Source*	*Identifier*
Acetic Acid, Glacial, Certified ACS	Fisher Scientific	A38-500
Acetone	Fisher Scientific	HC3001GAL
Chloroform, Certified ACS	Fisher Scientific	C298-1
Dimethyl Sulfoxide, Certified ACS	Fisher Scientific	D128-500
100% Denatured Ethyl Alcohol	Fisher Scientific	HC8001GAL
95% Denatured Ethyl Alcohol	Fisher Scientific	HC11001GL
Glycerol	SIGMA	G9012-1L
L-Lysine Hydrochloride	Fisher Scientific	BP386-100
Sodium-meta-periodate 99%	Fisher Scientific	AC19838-1000
Sodium phosphate dibasic dihydrate	SIGMA	71505-250G
Sodium phosphate monobasic heptahydrate	SIGMA	S2429-250G
Paraformaldehyde 96%	Fisher Scientific	AC41678-5000
Permount	Fisher Scientific	SP15-500
Thionin Acetate, pure, high purity biological stain	Fisher Scientific	AC22984-0050
Sodium Acetate Trihydrate, ACS Reagent Grade	Fisher Scientific	RDCS0250500
Xylenes, Histological Grade	Fisher Scientific	X3P-1GAL
CP13 monoclonal antibody	Fisher Scientific	MN1020
Peroxidase AffiniPure Goat Anti-Mouse IgG (H+L)	Jackson Immuno Research	115-035-003
3'3-Diaminobenzidine	Vector Laboratories	SK-4100
Vectasian Elite ABC kit (standard)	Vector Laboratories	PK-6100

Supplementary Table T1. Reagents and sourcing: Reagents used, source and catalog #.

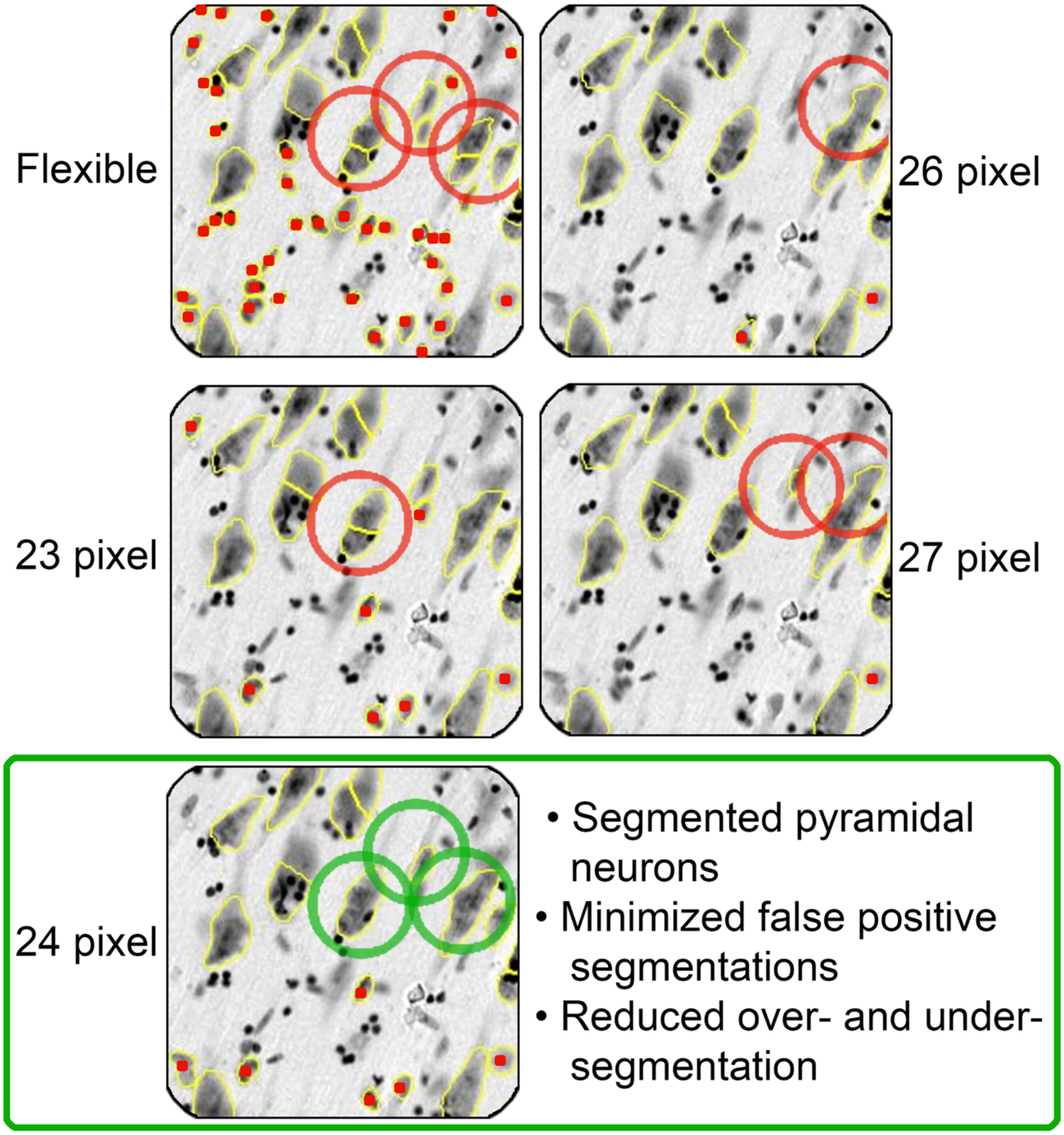

Supplementary Fig. F1. Identifying optimal CellPose pixel diameter input parameter. The panels show the piloting for the CellPose segmentation pixel diameter input parameter. The rows display different diameter input parameters (Flexible, 23 pixel, 24 pixel, 26 pixel, 27 pixel). Yellow outlines demarcate neuron segmentations from the extracellular matrix. Red dots indicate neuron profiles and glial cells (false-positives). Red circles mark instances of over- and under-segmentation of pyramidal neurons. Based on quality control evaluation, the CellPose input parameter of 24 pixel diameter demonstrated the best segmentation performance (green panel). It segmented pyramidal neurons, minimized false-positives segmentations, and reduced over- and under-segmentation (green circles).

Input parameter	*Value*
**model_type**	cyto
**pretrained_model**	true
**diameter**	24
**flow_threshold**	0.4
**mask_threshold**	0.0
**resample**	false
**use_gpu**	true
**save_png**	true

Supplementary Table T2. Input parameters used with the CellPose algorithm for pyramidal neuron segmentation in the human hippocampus.

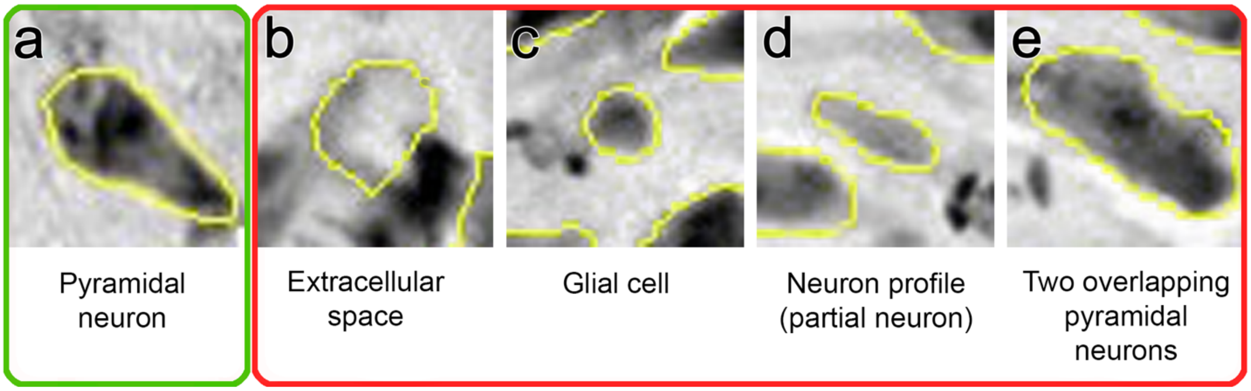

Supplementary Fig. F2. Segmented pyramidal neuron and false-positive segmentations. a) A pyramidal neuron segmented by CellPose is highlighted in the green panel. Four instances of false-positive segmentations were observed (red panel): b) segmented extracellular space, c) glial cells, d) neuron profiles (partial neurons), e) overlapping pyramidal neurons. To exclude these false-positive segmentations, a filtering method based on segmentation grey value and diameter was implemented.

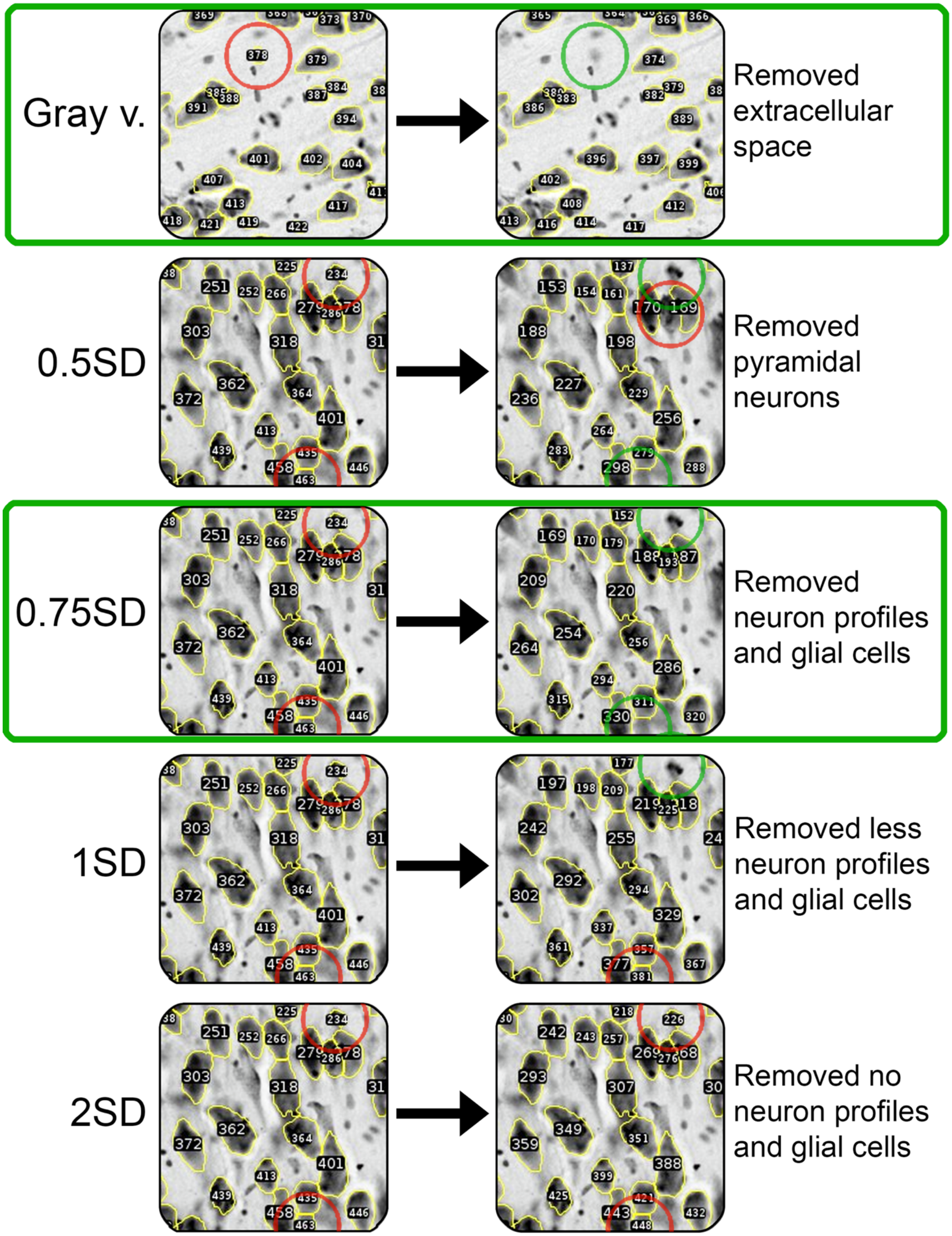
Gray v.
Removed extracellular space
0.5SD
Removed pyramidal neurons
0.75SD
Removed neuron profiles and glial cells
1SD
Removed less neuron profiles and glial cells
2SD
Removed no neuron profiles and glial cells

Supplementary Fig. F3. Identifying filtering parameters for the exclusion of false-positive hippocampal subregion pyramidal neurons. Various filters were piloted to assess optimal pyramidal neuron segmentations. In all panels, yellow outline shows neuron segmentations from the background. The rows display several filters: filtering based on mean gray value (Gray v.), and standard deviation filtering based on the average segmented pyramidal neuron diameter (0.5SD, 0.75SD, 1SD, 2SD). The first column shows the unfiltered segmentations, the second column the applied filter. Red circles demarcate false-positive segmentations. Green circles mark false-positive segmentations excluded by the implemented filters. Two filters (mean gray value and 0.75SD diameter) were found to effectively remove extracellular space, glial cells, and neuron profiles while preserving pyramidal neurons (green panel). These two filters were applied to the whole dataset.

Variable of interest, Unit	*Sub-region*	*n datapoints*	*25% Perc.*	*Median*	*75% Perc.*	*Mean*	*Std. Dev.*	*S.E.M.*	*Lower 95% CI*	*Upper 95% CI*
Filtered pyramidal neuron estimates, automated (CellPose), %	CA1	35	71.54	76.99	78.96	75.33	4.95	0.84	73.62	77.03
	CA1u	9	75.01	77.19	78.03	76.36	2.59	0.86	74.37	78.35
	CA2	21	75.88	77.42	78.35	77.18	1.63	0.36	76.44	77.92
	CA2u	11	75.44	77.77	78.33	77.24	1.49	0.45	76.25	78.24
	CA3	20	77.52	78.27	78.66	77.92	1.82	0.41	77.06	78.77
	CA3u	10	76.50	77.42	78.32	77.37	1.07	0.34	76.61	78.13
	CA4	16	71.21	74.80	77.35	73.69	4.37	1.09	71.36	76.02
	Sub	35	72.57	75.67	76.98	74.87	2.82	0.48	73.90	75.84
	Subu	12	73.59	77.36	78.24	76.34	2.76	0.80	74.59	78.10

Supplementary Table T2. Percentage of hippocampal pyramidal layer neurons remaining post filtering: 25% percentile, median, 75% percentile, mean, std. dev., S.E.M., lower 95% confidence interval and upper 95% confidence interval.

Variable of interest, Unit	*Sub-region*	*N datapoints*	*25% Perc.*	*Median*	*75% Perc.*	*Mean*	*Std. Dev.*	*S.E.M.*	*Lower 95% CI*	*Upper 95% CI*
Pyramidal neuron estimates, automated (CellPose), unfiltered	CA1	35	3255	5854	8605	6704.20	4440.90	750.66	5178.70	8229.70
	CA1u	9	1309	1768	3137	2189.10	1164.40	388.15	1294	3084.20
	CA2	21	439	584	763	619.33	196.93	42.97	529.69	708.97
	CA2u	11	1019	1178	1533	1281.50	473.58	142.79	963.39	1599.70
	CA3	20	832.25	1328	1621.30	1382.60	667.25	149.20	1070.30	1694.80
	CA3u	10	1514.80	2274	3045.50	2186.80	1017.50	321.77	1458.90	2914.70
	CA4	15	1235	1378	1749	1728.90	885.65	228.67	1238.40	2219.30
	Sub	35	3827	4739	7951	5985.10	3442.10	581.83	4802.60	7167.50
	Subu	12	2698.80	5916.50	6923.50	5426	2633.90	760.35	3752.50	7099.50

Supplementary Table T3. Unfiltered descriptive statistics of pyramidal layer neuron estimates per hippocampal subregion: 25% percentile, median, 75% percentile, mean, std. dev., S.E.M., lower 95% confidence interval, and upper 95% confidence interval of unfiltered automated pyramidal neuron estimates created by CellPose.

	Parameters	*n datap.*	*25% Perc.*	*Median*	*75% Perc.*	*Mean*	*Std. Dev.*	*S.E.M.*	*Lower 95% CI*	*Upper 95% CI*
Automated (CellPose) vs manual masks	Total overlap	15	0.79	0.84	0.85	0.83	0.05	0.01	0.80	0.85
	Jaccard score	15	0.39	0.50	0.61	0.49	0.12	0.03	0.43	0.56
	Dice score	15	0.56	0.66	0.76	0.65	0.11	0.03	0.59	0.71
	Volume similarity	15	0.16	0.43	0.64	0.42	0.30	0.08	0.25	0.58
	False-negative error	15	0.15	0.16	0.21	0.17	0.05	0.01	0.15	0.20
	False-positive error	15	0.30	0.45	0.57	0.44	0.15	0.04	0.36	0.53
Manual vs manual masks	Total overlap	15	0.44	0.69	0.81	0.64	0.19	0.05	0.54	0.75
	Jaccard score	15	0.38	0.49	0.56	0.48	0.10	0.03	0.42	0.53
	Dice score	15	0.55	0.66	0.72	0.64	0.01	0.03	0.58	0.69
	Volume similarity	15	-0.42	-0.15	0.33	-0.09	0.43	0.11	-0.33	0.15
	False-negative error	15	0.19	0.31	0.56	0.36	0.19	0.05	0.25	0.46
	False-positive error	15	0.21	0.31	0.41	0.31	0.11	0.03	0.25	0.38

Supplementary Table T4. Descriptive statistics for label overlap measures of automated vs manual masks, and manual vs manual masks: For five vignettes individually, three manual masks were compared pairwise and with one automated mask created using CellPose, resulting in 15 pairwise comparisons per group. 25% percentile, median, 75% percentile, mean, std. dev., S.E.M., lower 95% confidence interval, and upper 95% confidence interval are presented for the parameters of 1) total overlap, 2) jaccard score, 3) dice score, 4) volume similarity, 5) false-postive error, 6) false negative error. There was no significant difference between groups in Dice scores (Paired Samples t Test: $t(28) = 0.33$, $p = .742$).

	cases	*age, years ± SD*	*Pyramidal neuron counts, mean ± SD*
West and Gundersen (1990)	n = 5	66.8 ± 15.2	CA1 = 16.4x10^6 ± 5.1<br>CA2 + CA3 = 2.7x10^6 ± 0.6<br>Sub = 4.51x10^6 ± 0.9
Simić (1997)	n = 10	80.2 ± 7.9	CA1 = 11.8x10^6 ± 2.1<br>CA2 + CA3 = 2.5x10^6 ± 0.3<br>Sub = 4.2x10^6 ± 1.1

Supplementary Table T5. Pyramidal neuron counts in the hippocampal subregion reported by West and Gundersen (1990) and Simić (1997): West and Gundersen (1990) and Simić (1997) found that the hippocampal subregion CA1 had the highest number of pyramidal neurons, followed by subiculum, and combined CA2 and CA3. Both studies were performed on cognitive controls.
